# Central Serous Chorioretinopathy: Multimodal Imaging and Management Options

**DOI:** 10.1155/2020/8890404

**Published:** 2020-08-14

**Authors:** Prashanth G. Iyer, Stephen G. Schwartz, Jonathan F. Russell, Harry W. Flynn

**Affiliations:** Department of Ophthalmology, Bascom Palmer Eye Institute, University of Miami Miller School of Medicine, Miami, Florida, USA

## Abstract

Central serous chorioretinopathy (CSCR) is an idiopathic maculopathy characterized by thickened choroid, retinal pigment epithelial detachment, and variable subretinal fluid. CSCR predominantly affects young men, with risk factors including corticosteroid use, the type A behavior pattern, and psychological stress. While usually self-limited with a good visual prognosis, recurrent and persistent CSCR can lead to outer retinal and/or retinal pigment epithelial atrophy, choroidal neovascularization, and visual loss. This article reviews current multimodal imaging and treatment options, which include observation, mineralocorticoid receptor antagonists, thermal laser photocoagulation, and off-label photodynamic therapy with verteporfin.

## 1. Introduction

Central serous chorioretinopathy (CSCR) is a macular disorder thought to be due to alterations of the choroidal vasculature that overwhelm the ability of the retinal pigment epithelial (RPE) to dehydrate the subretinal space, with consequent accumulation of subretinal fluid. [1–3] Thickened choroid, RPE detachments, and variable subretinal fluid are cardinal features of CSCR. Patients may be asymptomatic or may complain of central scotoma, metamorphopsia, dyschromatopsia, and micropsia [4–6].

CSCR generally affects individuals between ages 30 and 50 years, with a predominance for men [7–11]. CSCR is most commonly seen in Asian and Caucasian patients [8]. Other risk factors include corticosteroid exposure, the type A behavior pattern (including a preponderance of a competitive drive, a sense of urgency, an aggressive nature, and a hostile temperament), psychological stress, *Helicobacter pylori* infection, use of phosphodiesterase 5 inhibitors, exogenous testosterone supplementation, obstructive sleep apnea, pregnancy, and some endocrine disorders [6, 11–19].

## 2. Clinical Features

Patients with acute CSCR typically present with clear and well-demarcated subretinal fluid. There is typically a smooth pigment epithelial detachment (PED) underlying the subretinal fluid, although the PED can be small and challenging to identify with biomicroscopy alone. The clinical course of CSCR is usually self-limited, with spontaneous resolution of subretinal fluid and restoration of visual acuity by three months [20]. The presence of turbid subretinal fluid and/or blood should alert the physician to the possibility of another diagnosis, including idiopathic choroidal neovascularization (CNV), exudative age-related macular degeneration, or polypoidal choroidal vasculopathy.

Some patients with CSCR will have a persistent or recurrent course beyond three months. Chronic CSCR can involve multifocal or diffuse RPE disruption and atrophy throughout the posterior pole, potentially causing severe visual loss [8]. RPE atrophy, along with outer retinal atrophy, secondary CNV, cystoid macular edema (CME), and choriocapillaris atrophy are causes of permanent visual loss [20] [21].

## 3. Imaging Modalities

### 3.1. Fluorescein Angiography

**Figure 1:**
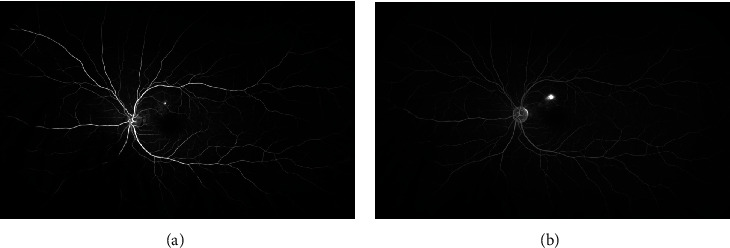
Fluorescein angiography with focal leakage, sometimes called an expansile dot. (a) Early phase. (b) Late phase.

**Figure 2:**
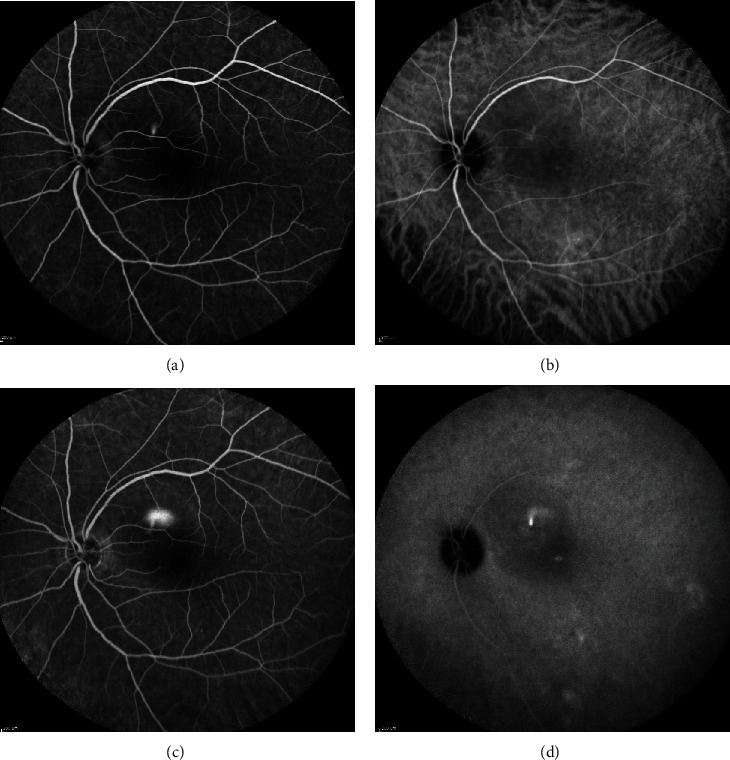
Simultaneous fluorescein angiography and indocyanine green angiography with a smokestack pattern. Early (a, b) and late (c, d) phase images are shown.

Fluorescein angiography (FA) can reveal various patterns in acute CSCR including focal leakage (sometimes called expansile dot) in the majority of cases (Figure 1) and a smokestack pattern in 10-20% of patients (Figure 2) [22].

Chronic CSCR may show multiple areas of mottled hyperfluorescence, indicating widespread patchy RPE dysfunction and/or atrophy [23]. FA can also detect the presence of secondary CNV.

### 3.2. Indocyanine Green Angiography

Indocyanine green angiography (ICGA) is another dye-based test that can detect impaired choroidal circulation in CSCR, including choroidal hypoperfusion, delays in choroidal circulation, and hyperfluorescence corresponding to areas of choroidal hyperpermeability [24, 25].

### 3.3. Fundus Autofluorescence

**Figure 3:**
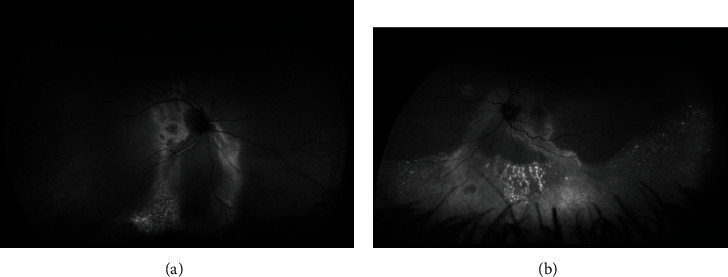
Fundus autofluorescence of two different patients with chronic central serous chorioretinopathy with descending tracts or “guttering”.

Fundus autofluorescence (FAF) detects the endogenous fluorophores found in lipofuscin of the RPE cells, providing information regarding the health of the RPE [26, 27]. FAF patterns in acute CSCR may include confluent or granular hypoautofluorescence signifying loss of RPE cells and hyperautofluorescence representing accumulation of unphagocytized photoreceptor outer segments [28]. In chronic CSCR, there is multifocal or diffuse mottled hypoautofluorescence representing atrophy of the RPE. There can be areas of hyperautofluorescence. Descending tracts, also known as "guttering", (Figure 3) with increased and decreased autofluorescence may be seen [28, 29].

### 3.4. Optical Coherence Tomography

**Figure 4:**
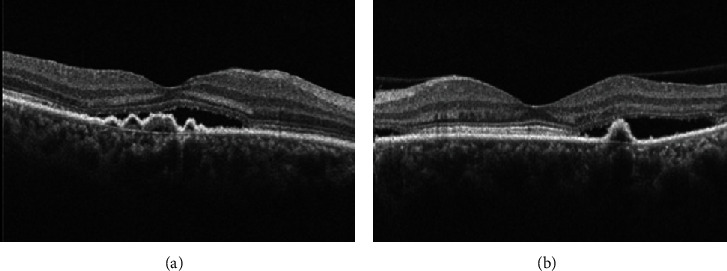
Optical coherence tomography in central serous chorioretinopathy. Note multiple pigment epithelial detachments, subretinal fluid, and “shaggy” photoreceptors.

**Figure 5:**
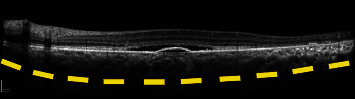
Optical coherence tomography in central serous chorioretinopathy. Note the pigment epithelial detachment and subretinal fluid. Enhanced depth imaging shows a thickened choroid (yellow dashes indicate the sclerochoroidal junction).

Optical coherence tomography (OCT) allows for rapid noninvasive identification of PED, CME, subretinal fluid, and other structural abnormalities (Figure 4). In addition, enhanced depth imaging OCT can detect increased choroidal thickness, which is characteristic of CSCR [30] (Figure 5). CSCR can be described in the spectrum of pachychoroid diseases, which share similar features of choroidal thickening and retinal pigment epithelial changes. [21, 31–33] The exact clinical significance of the different pachychoroid disorders with respect to treatment outcomes is uncertain and still under investigation [34].

### 3.5. Optical Coherence Tomography Angiography

Optical coherence tomography angiography (OCT-A) is a noninvasive, non-dye-based technique of angiography that can identify vascular flow in the different layers of the retina and choroid. OCT-A in CSCR can detect choroidal and choriocapillaris deficits, underlying CNV, and simultaneously includes B-scans that may feature PED, subretinal fluid, CME, and outer retinal and/or RPE atrophy [35–37].

## 4. Management

### 4.1. Observation

**Figure 6:**
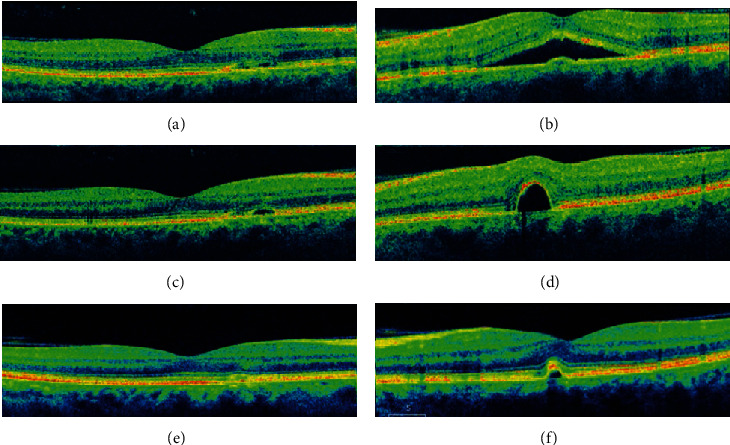
Observation of a patient with bilateral central serous chorioretinopathy. A 52-year-old male with a history of oral corticosteroid use presented with many months of blurred vision, left eye worse than the right eye. The patient was observed every few months without therapy. (a, b) Right and left eye (respectively) optical coherence tomography scans at baseline revealed subretinal fluid and pigment epithelial disruptions. (c, d) One year later, the left eye subretinal fluid had resolved but the pigment epithelial detachment was more elevated. (e, f) Two years later, the subretinal fluid had resolved in both eyes with mild pigment epithelial disruptions. Visual acuity remained stable at 20/20 in each eye throughout this timeframe.

**Figure 7:**
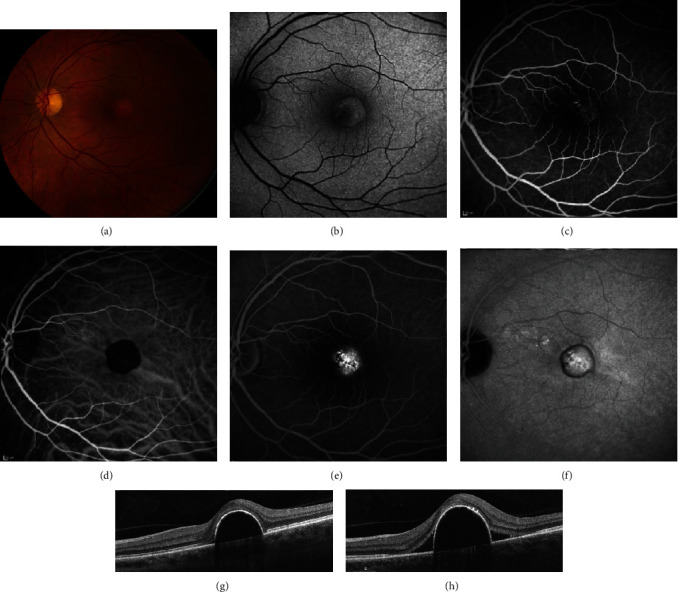
Central serous chorioretinopathy of the left eye. A 56-year-old male was observed for 3 years with worsening of metamorphopsia. (a) Fundus photography showed pigment irregularities and clumping over a pigment epithelial detachment (PED). (b) Fundus autofluorescence showed hypoautofluorescent changes surrounding the PED with granular hyperautofluorescence centrally. (c) Early-phase fluorescein angiography (FA) revealed faint pinpoint leakage and staining of the PED. (d) Early-phase indocyanine green angiography (ICGA) showed blockage due to the PED. (e, f) Late-phase FA and ICGA showed macular leakage. (g) Optical coherence tomography (OCT) showed a PED but no subretinal fluid. (h) One year later, OCT showed worsening of the PED and new subretinal fluid.

The majority of patients with CSCR will have spontaneous resolution of subretinal fluid with recovery of vision. Observation is the first-line therapy for most patients who have had symptoms for less than about three months [5, 21] (Figures 6 and 7). The use of exogenous corticosteroids should be reduced or discontinued if possible. Occasionally, treatment can be initiated sooner for patients requiring more rapid improvement in vision for occupational or other needs.

### 4.2. Thermal Laser Photocoagulation

**Figure 8:**
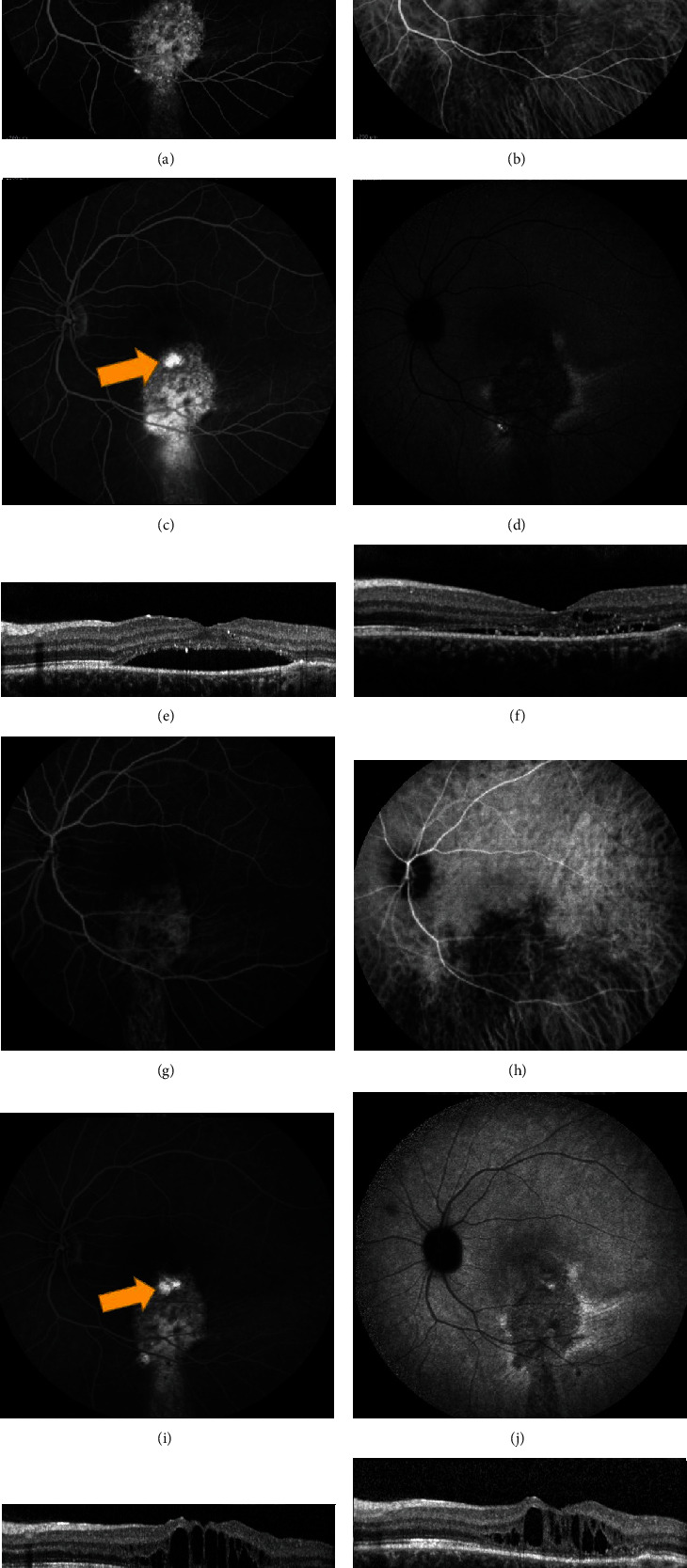
Chronic central serous chorioretinopathy treated with thermal laser photocoagulation. A 21-year-old female presented with visual distortion for over one year. Visual acuity in the left eye was 20/150 at presentation. (a–f) Images at baseline. (a) Early-phase fluorescein angiography (FA) showed a broad area of hyperfluorescence. (b) Early-phase indocyanine green angiography (ICGA) showed areas of hypofluorescence corresponding to the hyperfluorescence on FA. (c) Late-phase FA showed extrafoveal focal leakage, noted by the yellow arrow. (d) Late-phase ICGA showed hyperfluorescence corresponding to the focal leak on FA. Thermal laser was applied to the area corresponding to the leakage on FA and ICGA. Visual acuity improved to 20/100. (e) Optical coherence tomography (OCT) prior to thermal laser showed subretinal fluid and “shaggy” photoreceptors. (f) Post thermal laser, there was improvement in subretinal fluid. The patient was lost to follow-up but returned three years later with worsening symptoms. Visual acuity was 20/800. (g) Early-phase FA showed broad hyperfluorescence. (h) Early-phase ICGA showed hypofluorescence. (i) Late-phase FA showed two small areas of extrafoveal focal leakage, noted by the yellow arrow. (j) Late-phase ICGA showed one area of focal hyperfluorescence corresponding to the leakage on FA. (k) OCT prior to repeat thermal laser treatment showed loss of outer retinal layers with recurrent intraretinal fluid. (l) OCT following the second argon photocoagulation (yellow arrow in (i)) showed improvement in fluid as well as macular thickness. Visual acuity returned to 20/150 after treatment.

**Figure 9:**
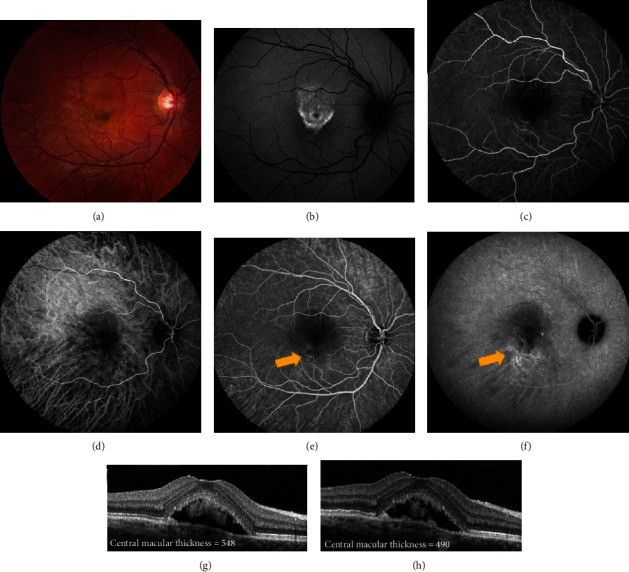
Chronic central serous chorioretinopathy treated with thermal photocoagulation. A 17-year old male presented with blurred vision of the right eye for nine months. Visual acuity was 20/50 in the right eye. (a) Fundus photography showed pigment changes and subretinal fluid in the right eye. (b) Fundus autofluorescence showed hyperautofluorescence of the macula, corresponding to the subretinal fluid seen clinically. (c, d) Early-phase fluorescein angiography (FA) and indocyanine green angiography (ICGA) showed focal hyperfluorescence corresponding to the inferior edge of the fluid seen clinically. (e) Late-phase FA showed leakage in the inferior macula, noted by the yellow arrow. (f) Late-phase ICGA confirmed the leakage, noted by the yellow arrow. Thermal photocoagulation was chosen because of the young age and extrafoveal focal leakage. Yellow arrows indicate where thermal laser was applied. (g, h) Optical coherence tomography before (g) and one month after (h) thermal photocoagulation showed decrease in subretinal fluid (measured in *μ*m) and improvement in central macular thickness. Visual acuity improved to 20/40+. The patient was then lost to follow-up.

Thermal laser photocoagulation is a well-established option for treating CSCR by directly applying argon laser to a leaking site seen on FA and/or ICGA (Figures 8 and 9). Thermal laser photocoagulation has been reported to decrease subretinal fluid and improve visual acuity [38]. However, long-term visual outcomes have not been reported to improve significantly with argon laser treatment [39, 40]. While most studies reported no differences in recurrence rates associated with laser photocoagulation, there are a few studies that reported lower rates of recurrence in laser-treated patients [41–44]. Complications of thermal laser photocoagulation include scar formation and secondary CNV. Given the potential for scarring, argon laser is most appropriate for extrafoveal leaking sites [42].

Subthreshold micropulse laser was initially described by Bandello and colleagues. In their pilot study, five patients with chronic CSCR were treated and had complete subretinal fluid resorption without recurrence; follow-up data and subsequent case studies have been promising [45–47].

### 4.3. Photodynamic Therapy

**Figure 10:**
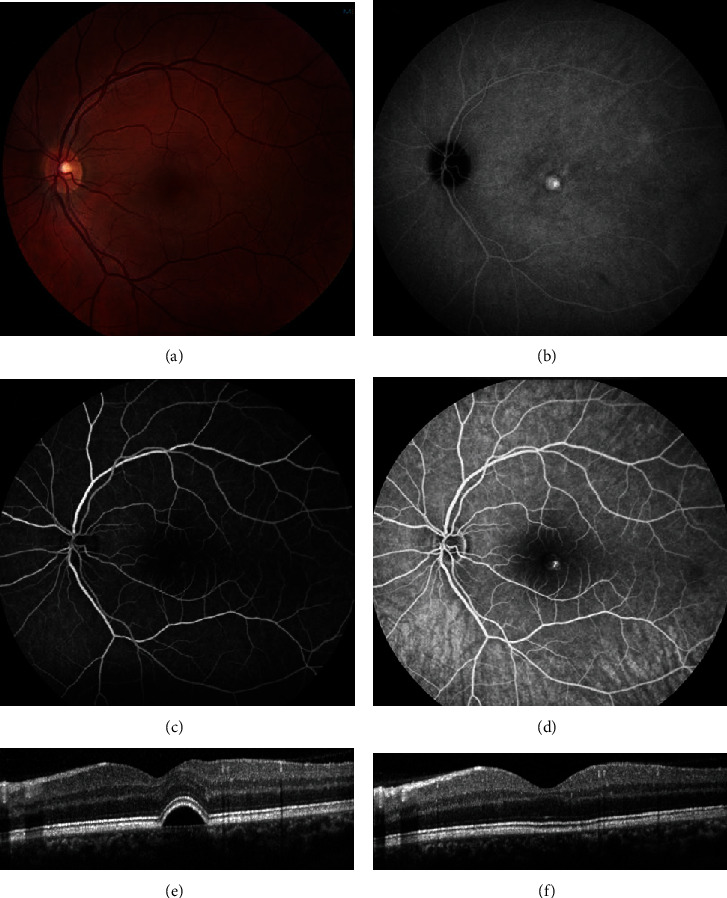
Chronic central serous chorioretinopathy treated with photodynamic therapy (PDT). A 35-year old male presented with 6 months of visual distortion in the left eye, although visual acuity was 20/20. (The amount of metamorphopsia was not formally measured.) He had tried 50 mg of eplerenone daily for over one month without improvement. (a) Fundus photography showed pigment changes and a pigment epithelial detachment (PED). (b) Fundus autofluorescence showed an area of hyperautofluorescence corresponding to the PED. (c) Early-phase fluorescein angiography (FA) showed a faint area of leakage in the macula. (d) Late-phase FA showed increased leakage in the macula. (e) Optical coherence tomography prior to and (f) after half-fluence PDT showed resolution of the PED. Visual acuity remained 20/20 and the patient reported subjective improvement.

**Figure 11:**
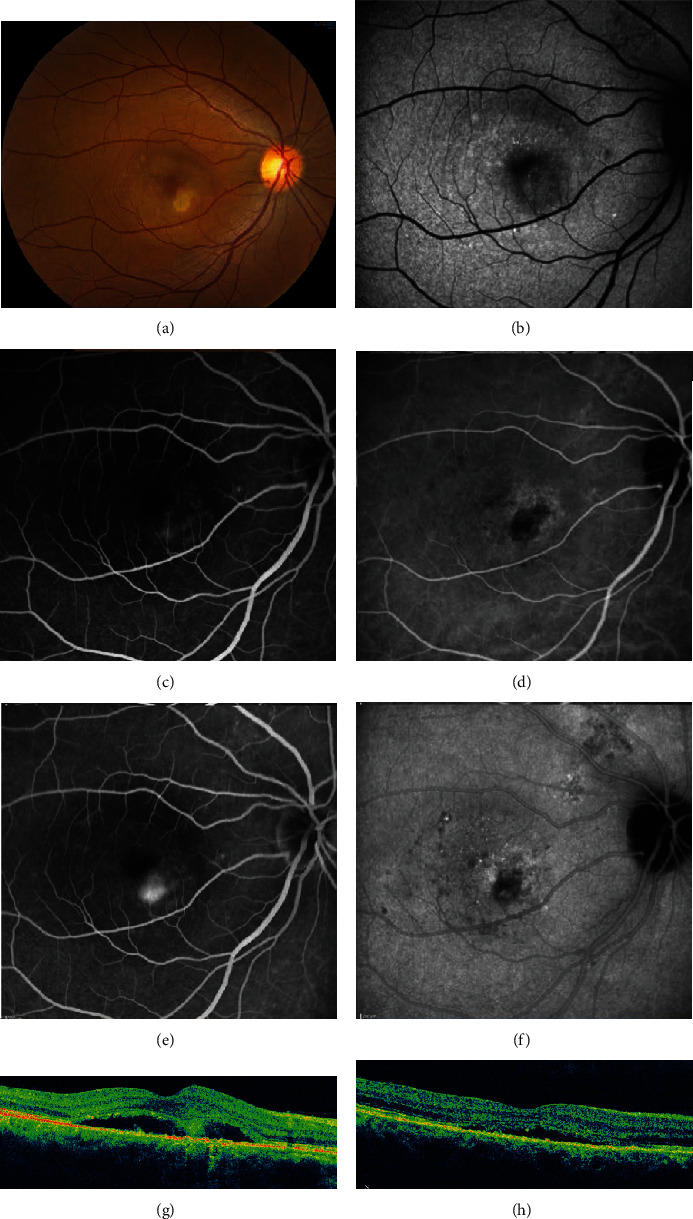
Chronic central serous chorioretinopathy of the right eye treated with reduced fluence photodynamic therapy (PDT). A 49-year old female presented with blurred vision in the right eye. Visual acuity was 20/50. (a) Fundus photography showed pigmentary changes of the right eye. (b) Fundus autofluorescence showed hypoautofluorescence at the macula. (c, d) Early-phase fluorescein angiography (FA) and indocyanine green angiography (ICGA) showed hyperfluorescence. (e, f) Late-phase FA and ICGA showed leakage in the inferior macula. (g) Optical coherence tomography prior to and (h) after half-fluence PDT showed improvement of subretinal fluid. Visual acuity improved to 20/25.

**Figure 12:**
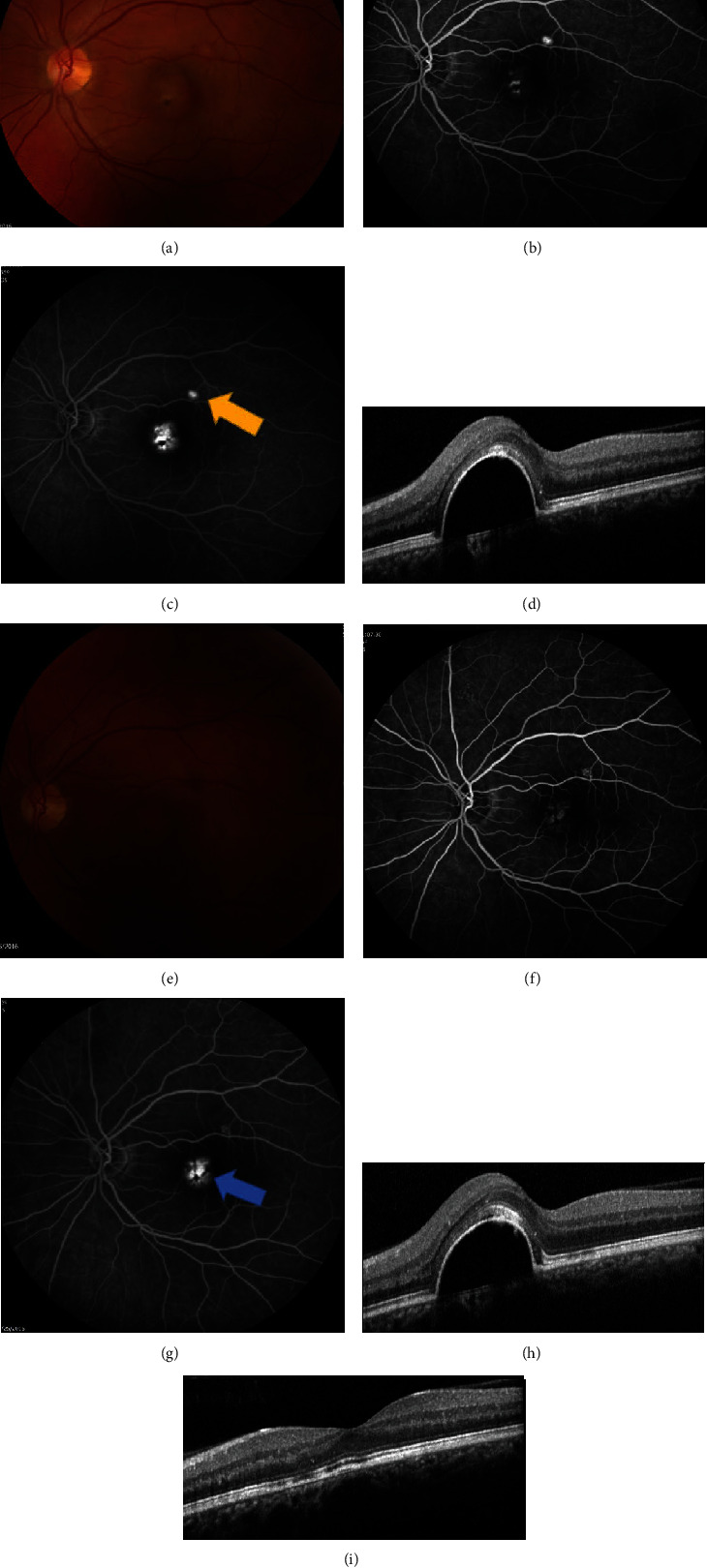
Chronic central serous chorioretinopathy of the left eye treated with both thermal laser photocoagulation and half fluence photodynamic therapy (PDT). A 42-year old female presented with blurred vision for two years. (a–d) Images at baseline. (a) Fundus photography showed pigmentary changes around a pigment epithelial detachment (PED). (b) Early-phase fluorescein angiography (FA) showed leakage in the fovea as well as slightly superotemporal. (c) Late-phase FA showed increased leakage; the superotemporal lesion is noted by the yellow arrow. (d) Optical coherence tomography (OCT) showed a large subfoveal PED. The patient was initially treated with thermal photocoagulation to the superotemporal lesion (yellow arrow in (c)), so as to spare the fovea. (e) Following thermal photocoagulation, the clinical appearance did not change. (f) Early-phase FA and (g) late-phase FA showed improvement of the treated superotemporal lesion but persistent leakage of the untreated subfoveal PED. (h) OCT showed no change in the subfoveal PED (noted by the blue arrow in (g)). The patient then underwent half-fluence PDT (blue arrow in (g)). (i) One month later, OCT showed resolution of the pigment epithelial defect. Visual acuity improved from 20/30 to 20/20.

Off-label photodynamic therapy (PDT) with verteporfin (Visudyne, Bausch + Lomb, Bridgewater, NJ) was first described in a case series of 15 patients with chronic CSCR. Complete resolution of subretinal fluid occurred in 12 of the 15 patients [48]. PDT has been reported effective in resolving subretinal fluid and improving visual acuity [48–50]. Complications associated with PDT include secondary CNV, choriocapillaris hypoperfusion, and RPE damage [51]. In an effort to reduce complications, low dose verteporfin and lower fluence PDT have been reported effective with lower risks of adverse effects [52, 53]. Half-dose PDT has been reported to reduce subretinal fluid compared to argon laser in CSCR; however, visual acuity outcomes were similar in both groups [40] (Figures 10–12). The authors are anecdotally aware of, but have no personal experience with, the use of PDT in pediatric patients.

### 4.4. Eplerenone and Spironolactone

Eplerenone and spironolactone are two mineralocorticoid receptor (MR) antagonists that are increasingly used in the off-label treatment of CSCR. One hypothesis for the etiology of CSCR is inappropriate activation of MR by glucocorticoids; of note, corticosterone induces choroidal dilation, hyperpermeability, and egress of fluid across the RPE into the subretinal space [54]. Spironolactone is a nonselective antagonist of the aldosterone receptor. Its effects of blocking the aldosterone receptor are hypothesized to decrease choroidal hyperpermeability from off-target binding of corticosteroids. Spironolactone has multiple effects on estrogens and androgens that can result in unwanted side effects in male patients such as gynecomastia, decreased libido, and erectile dysfunction [55–57].

Eplerenone is a newer, more selective aldosterone antagonist that binds to the MR and less to the other steroid receptors. Eplerenone has a 10- to 20-fold lower affinity to MR, and a 100- to 1000-fold lower affinity to the other steroid receptors than spironolactone, resulting in fewer side effects [57, 58]. In general, both MR antagonists are prone to hyperkalemia and potassium levels should be monitored [55]. The authors have no personal experience with the use of MR antagonists in pregnancy or in pediatric patients.

**Figure 13:**
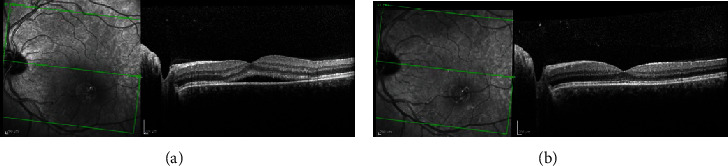
Central serous chorioretinopathy treated with eplerenone. A 44-year old Hispanic male presented with distortion in left eye although visual acuity was 20/20. He reported symptoms for three months. (a) Optical coherence tomography (OCT) confirmed subretinal fluid. The patient was started on eplerenone 25 mg daily. (b) One month after starting treatment, OCT showed resolution of the subretinal fluid. Subjectively, the patient reported improvement.

Bousquet et al. conducted a prospective, nonrandomized study of 13 patients with CSCR symptomatic for at least four months. These patients were treated with 25 mg of oral eplerenone daily for one week, followed by 50 mg daily for 1-3 months. Treatment with eplerenone was associated with significant improvements in central macular thickness, subretinal fluid, and visual acuity [59]. The authors followed up with a prospective, randomized, double-masked, placebo-controlled study of 16 patients randomized to receive 50 mg of spironolactone or placebo daily for 30 days. There was a significant reduction of subretinal fluid and subfoveal thickness in the treatment arm compared to placebo; however, no significant changes in best-corrected visual acuity were reported [60]. Herold et al. reported another prospective, nonrandomized study in 18 patients with chronic CSCR evaluating spironolactone 25 mg twice daily for 12 weeks. They reported improvements in subretinal fluid, central macular thickness, and visual acuity [61] (Figure 13).

### 4.5. Anti-vascular Endothelial Growth Factor Therapy

**Figure 14:**
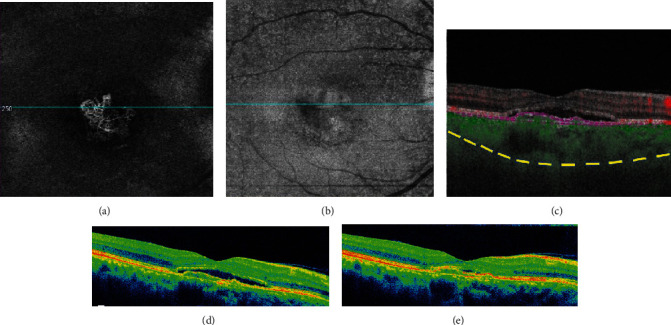
Chronic central serous chorioretinopathy of the right eye with secondary choroidal neovascularization (CNV). A 53-year old female with chronic central serous chorioretinopathy presented with worsening subretinal fluid. Using swept source optical coherence tomography angiography (SS-OCTA–PLEX Elite 9000, Carl Zeiss Meditec, Dublin, CA) 6 × 6 scans were performed; shown is the slab between the retinal pigment epithelium (RPE) and Bruch's membrane. (a) En face angiography showed the presence of type 1 choroidal neovascularization (CNV). (b) En face structural imaging showed the RPE elevation due to underlying CNV. (c) B-scan demonstrated subretinal fluid. The presence of a pigment epithelial detachment was seen, with increased flow (green) within the detachment indicating increased choroidal flow in the presence of CNV. The sclerochoroidal junction (yellow dashes) was highlighted to demonstrate the thick choroid. OCT prior to (d) and after (e) anti-vascular endothelial growth factor injections showed improvement of subretinal fluid.

**Figure 15:**
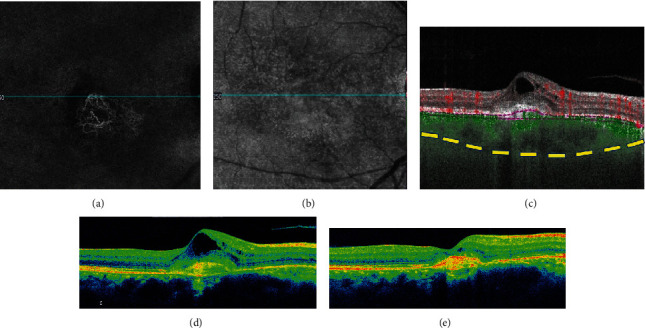
Chronic central serous chorioretinopathy of the right eye with secondary choroidal neovascularization (CNV). A 59-year old male with a history of inhaled triamcinolone use for asthma presented with worsening vision. Using swept source optical coherence tomography angiography (SS-OCTA – PLEX Elite 9000, Carl Zeiss Meditec, Dublin, CA), 6 × 6 scans were analyzed; shown is the slab between the retinal pigment epithelium (RPE) and Bruch's membrane. (a) En face angiography showed the presence of type 1 choroidal neovascularization. (b) En face structural imaging showed RPE elevation causing shadowing. (c) B-scan demonstrated cystoid macular edema, with increased flow (green) indicating increased choroidal flow in the presence of CNV. The sclerochoroidal junction (yellow dashes) was highlighted to demonstrate the thick choroid. (d, e) OCT prior to and after anti-vascular endothelial growth factor injections showed improvement of intraretinal fluid and hyperreflective material.

Anti-vascular endothelial growth factor (anti-VEGF) therapy is mainly used for chronic CSCR with secondary CNV (Figures 14 and 15). In cases without CNV, there is no evidence of elevated VEGF levels in plasma or aqueous, suggesting that anti-VEGF agents would be ineffective for the treatment of acute or chronic CSCR [62]. However, a few case series have reported variable efficacy using anti-VEGF agents for patients with chronic CSCR and no apparent CNV [63–65].

## 5. Conclusions

Acute CSCR is often self-limiting, so treatment of CSCR is generally not indicated except when symptoms persist for more than about 3 months. Some patients, especially monocular patients or patients with specific occupational needs, may benefit from earlier intervention. Chronic, nonresolving CSCR has no widely accepted and definitive treatment, due to the paucity of head-to-head clinical trials. In such cases, the authors prefer to use combined FA and ICGA to identify extrafoveal sites of leakage that might be amenable to thermal photocoagulation, which is safe to use in almost all patients, including pregnant patients and in pediatric patients.

**Figure 16:**
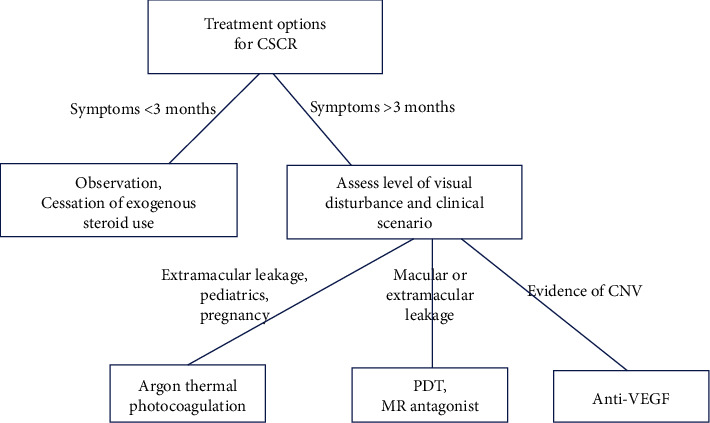
Review of treatment options in patients with central serous chorioretinopathy (CSCR). CNV: choroidal neovascularization; MR: mineralocorticoid; PDT: photodynamic therapy; VEGF: vascular endothelial growth factor.

If there is no focal extrafoveal leak on angiography, options other than thermal photocoagulation include reduced fluence PDT and/or MR antagonists. This is typically an individualized decision. Some patients may be more willing to take a long-term systemic medication rather than undergo PDT with its required period of sunlight avoidance; other patients may prefer the reverse.  Figure 16 summarizes the treatment options in CSCR.
